# Human Dissection in Medical Education: More than Just Anatomy

**DOI:** 10.3205/zma001067

**Published:** 2016-11-15

**Authors:** Gerd Rehkämper

**Affiliations:** 1 Heinrich Heine Universität Düsseldorf, Universitätsklinikum, Institut für Anatomie I, Düsseldorf, Germany

**Keywords:** dissection course, values, concept of humans, competencies, ethics

## Abstract

The dissection course is an essential component of the medical curriculum. Nonetheless, the time expenditure and intensity of supervising the students in this course has been diminishing since the 1970s. That endangers not only the transmission of fundamental knowledge of anatomy. It also concerns key concepts such as **establishing values, the concept of humans**, and** physician competencies**, because medical education must be seen not merely as fact-directed instruction but instead should be connected with a mission for professional acculturation.

## Zusammenfassung

Der Präparierkurs ist ein essentieller Bestandteil des Medizinstudiums; ungeachtet dessen verringern sich seit den 70er Jahren Zeitaufwendung und Intensität der studentischen Betreuung. Das gefährdet nicht nur die Vermittlung anatomischer Grundkenntnisse. Es betrifft auch Stichworte wie **Wertsetzung, Menschenbild** und **ärztliche Kompetenzen**, weil das Medizinstudium nicht nur als faktengeleitete Lehre gesehen werden muss, sondern mit einem Bildungsauftrag verbunden sein sollte.

## 1. Problems and Possibilities of the Dissection Course

The dissection course in an essential part of medical education [22], [24], [34], [48]. But its status has changed over time. In the Appointment Order (1953), a two-semester dissection course was planned [http://www.bgbl.de/xaver/bgbl/start.xav?startbk=Bundesanzeiger_BGBl&jumpTo=bgbl153s1334.pdf]. Through the Approbation Order (1970) [https://www.uni-mainz.de/studlehr/ordnungen/AppO_Medizin_bis_09_03.pdf], [39], the course became *de facto* one semester. This halving of the course time was criticized [50] as much as the generally (too) short designated time for dissection [24], [30]. This tendency toward “concentration” of the course has intensified. The orientation toward obtaining external funding forces professors and other staff to invest as much time as possible in research and to pull back from teaching duties. Additional engagement in teaching beyond what is required is hardly possible. Increasing numbers of students yet reduced numbers of scientific staff aggravate the situation further.

Also in regards to the content, the dissection course and macroscopic anatomical teaching has become delimited. Whereas earlier textbooks [5] were still able to transmit causal-analytic ways of thinking, new textbooks [2] must often limit themselves to the purely factual. Macroscopic anatomy is threatened to deteriorate into a pure memorization stunt [35].

The dissection course has been criticized fundamentally [28], [29]. But there was strong dissent against the critiques and calls for its preservation with warnings against its further shortening [24], [7], [27], [35], [46]. I would like to join these defenses but also emphasize that the dissection course can transmit much more than only macroscopic-anatomical knowledge. It helps to develop **competencies, values**, and a** concept of humans**, which lies at the basis of the self-understanding of the physician and his or her view of the patients [8], [38], [51]. 

My exposition should show the connection between the course and the formation of competencies, values, and concepts of humans. I will not follow a linear argument, and I do not aim to present an overview article. Rather, the key word “dissection course” is the means and/or starting point to show, in a spotlighting way, how it can be the initial point to train competencies, values, and concepts of humans.

## 2. The Beginning of the Course

Due to its position at the beginning of the medical curriculum, the course is linked to a psychological challenge, which is mostly well mastered and even strengthens the learning success [3], [10], [49]. When the course is mentally stressful, the students should not deal with that by themselves alone but should make the problem known in conversations or forums [1], [54]. That eases the processing for the student concerned and serves also the group. **Social competencies** are thereby promoted. 

Contact with a dead person leads to existential questions such as the finitude of human being (death) but also to the question of body donation and its meaning. That should be addressed. Thoughts about **death** are often repressed [13], [18]. Consciously perceiving it focuses one’s view of one’s own life and leads to self-reflection and **self-competency**. It also shows the limits of treatability [4], exhorting to **humility**. The latter protects the physicians from expectations that are too high, the non-fulfillment of which can be accompanied by loss of trust from the patient. Also the question of whether one should do everything medically possible to maintain life belongs here [19]. The physician must take a position toward life-prolonging measures [13], and think through the definition of “dead” [15]. The dissection course offers the first opportunity for that. 

Acceptance of anatomical dissection and **body donation** did not always exist [9], [33], [34], [48], [49], [52], [55]. The **Enlightenment** required rethinking; a scientific worldview is reflected therein [21], [26], [44], [47]. Making that clear to oneself leads to **human-biological and scientific competence**.

“Science” implies a causal-analytic **cause-effect thinking** with a process of falsification [24], [40], see below]. That is the basis of **diagnostic competence** [11]. Grappling with an “evidence-based medicine” proves the importance of a “scientifization” of medicine [42].

## 3. Values, Freedom, and Self-Determination of Humans

Value systems are the subject of ethics and typical for humans [47]. A person is born into a value system and should consciously implement its principles, especially since individuals are assigned a lot of self-responsibility nowadays [32], [43].

The dissection course shows an enlightened scientific thinking on the part of everyone (body donor, students, instructors). That is fundamental for the discussion of values. The viewpoint that scientific thinking must be carried out value-free [53] is surpassed [45]. The physician needs a value scale, in order to reach decisions. No legal system can take over for that, because there is leeway in physician decision-making [4], [41]. The scientific corpus of theory can help there.

The **theory of evolution** comes into play here. It is the theory of scientific disciplines [6], [16], [17], [31].** Individuality** and **responsibility** for the next generation are in its central concepts [31]. The dissection should therefore be used to talk about this, in order to found the **human-biological competence** of the physician.

The acquisition of **ethical competence** can begin in the dissection course.

## 4. Individuality and Integrity of Humans

Anatomy textbooks emphasize what is generally typical. Its reflection in the reality of the body donor accentuates the **individuality** of the person. That is closely tied to the **communicative competence** of the physician, who treats always an individual [24].

Ultimately, also our social life together is touched by “individuality”, which in turn requires a **social competence** as its basis. It is the answer to the question, whether the interests of the society stand above those of the individual person or vice versa. Our culture tries to find a middle road, whereby the physician in turn gives the individual a particular attention through anamnesis, diagnosis, and therapy.

The individuality of the person is shown also in his or her three-dimensionality. That is a challenge for scientific dissection. Scientific work means resolving a complex structure into less complex subunits, in order then to return to the complexity [20]. Here it counts to transmit to the students, through the dissection, the conscious recognition that the individual person confronts us in a **three-dimensional integrity**. An additional benefit arises because the corpse, which is anonymous at first, acquires an individual face through the analytic-synthetic process [24].

Health and sickness are a phenomena of the whole organism. The foundation for such a **holistic consideration** can be laid in the dissection course. The traditional dissection process [50] strives for a holistic consideration but could be further optimized, in order to make the aim clearer. One could, without loss of knowledge, largely forego the stripping off of body parts (extremities) or the removal of organs (upper abdominal packet, colon) through a somewhat more sophisticated dissection.

## 5. Functional (i.e. Scientific) Thinking

The dissection of muscles with origin and projection makes it possible to teach functional thinking. That is fundamental [11], [24]. Every diagnostic and therapy has as its goal to recognize and eliminate functional disorders. For some muscles it is easy to work out the function, because only one function is possible for them; others have primary and secondary functions. Yet an understanding of the function is always possible through the dissection of projection and origin and a reflecting consideration of the findings on the skeleton.

Thus one learns to think causally in cause (i.e. topographic situation) and effect (i.e. resulting movement) and becomes familiar with the heuristic basic principle of scientific work. That includes the experimental approach [40]. One can develop a hypothesis and make a small experiment, e.g. one pulls on a superficial sinew of the lower arm and thereby initiates a flexion in the proximal interphalangeal joint of the middle finger. Then it is clear that one is acting on the *Musculus flexor digitorum superficialis*, which is the hypothesis one had proposed.

Thus the dissection course leads through the dissection from simple to complex causal connections. The course thus offers a thought-training, which, embedded into a theoretical framework, teaches scientific competence (cf. also [24]).

Critics assert that too much scientificity leaves the physician acting without empathy toward the patient. But that is not a consequence of the experience of the dissection course [8].

## 6. Humans as a Result of a Developmental Process

The body of a person is something that has “become”. It has an ontogenesis (individual development) and a phylogenesis (history of origins). The macroscopic dimension of ontogenesis (“embryology”) often has a small place in the curriculum, unfortunately. The dissection course offers approaches to counteract that, particularly a multitude of treatable illnesses have an embryological background (e.g. heart defects). If one integrates findings of modern molecular embryology, comprehension is deepened and new therapy approaches are identified.

Phylogenetic aspects are often neglected. Comparative anatomy can show the degree to which man is especially differentiated (e.g. in the brain) and where hardly any raised level of differentiation is reached (e.g. in the digestive tract). That also defines a concept of humans; it belongs to the **“human-biological competence”**.

## 7. The Special Place of the Brain

No other organ is to “mystified” as the brain, so tightly related to the concepts of “personality” and “human being”, and so complex in its function. It is the “organ of the soul” of Soemmerring [14], and the influence of the “soul” on health and sickness is great. Even the question of the freedom of humans has received new stimulus from brain research [12]. Therefore study of the brain is allocated greater time in the dissection course. That also belongs to **“human-biological competence”**. So it is especially important during the dissection of the brain to discuss functional analytic methods (EEG, MRT, fMRT, PET, brain stimulation), in order to discern the connection between form and function. Accompanying seminars are suitable for this.

## 8. The Question of Time

Optimal conditions would be provided when a small group of students could discuss each step of the dissection with an experienced teacher and there were no pregiven time parameters. We are far removed today from such a “gold standard”.

“Free dissection” without the presence of an instructor could help. The prerequisite is that the students receive aid materials. The internet can take over an important role here, in which the dissection instructions, teaching films, and interactive offers are made available. That is indeed suboptimal, but it nonetheless offers a chance to attenuate the disadvantages of the current educational system.

The students must thereby develop an ability for self-organization. That leads to self-competence, which is fundamental for being a doctor anyway. So the course helps to make a virtue out of necessity.

## 9. The Commemoration

The students get to know “their” corpse and the life background of the donor well. So they want an appropriate farewell at the end of the course. The tradition of a commemoration has resulted from that [1], [37], [36]. It offers the chance of recollection, beyond the pressures of examination. The ceremony and its preparation are again the occasion for **(self-)reflection** [25]. They support seeing in the body donor a human being with inviolable dignity. One positions oneself thereby also against an effect-gimmicky exhibition of the person [23].

If the commemoration addresses itself to the relatives of the body donor, the students become mediators between science and the population. They may then succeed in convincing the relatives of the value of the body donation, because they believably demonstrate how important the donation was for them. A **communicative competence** is apparent therein.

## 10. Knowledge of Facts or Education or Both?

During the educational program, one learns a knowledge of facts, upon the basis of which an advanced education can take place, which improves the chances of healing. Yet the physician is not only a “healer” but also an advisor for many decisions important to life. That requires a value system and a concept of humans. Transmitting such a thing is connected with the concept of education and to this degree, the question posed can be answered with “both”. The dissection course offers a good platform for this, above all when it is well interlinked with other courses.

## Acknowledgements

I would like to give special thanks to Univ.-Prof. Dr. Reinhard Hildebrand (Münster) and Univ.-Prof. Dr. Gerd Novotny (Düsseldorf) for the stimulating exchange of ideas. I would also like to thank Michael Hanna, PhD, New York (USA), for translating the manuscript from German into English.

## Competing interstes

The author declares that he has no competing interests.
